# Metabolomics Study of Metabolic Changes in Renal Cells in Response to High-Glucose Exposure Based on Liquid or Gas Chromatography Coupled With Mass Spectrometry

**DOI:** 10.3389/fphar.2019.00928

**Published:** 2019-08-20

**Authors:** Liang Wang, Yan Du, Bing-ju Xu, Xu Deng, Qing-hua Liu, Qiao-qiao Zhong, Chen-xiang Wang, Shuai Ji, Meng-zhe Guo, Dao-quan Tang

**Affiliations:** ^1^Jiangsu Key Laboratory of New Drug Research and Clinical Pharmacy, School of Pharmacy, Xuzhou Medical University, Xuzhou, China; ^2^Department of Bioinformatics, School of Medical Informatics, Xuzhou Medical University, Xuzhou, China; ^3^Deparment of Pharmaceutical Analysis, School of Pharmacy, Xuzhou Medical University, Xuzhou, China

**Keywords:** dynamic metabolomics, diabetic nephropathy, diabetes mellitus, renal cell, biomarker identification

## Abstract

Diabetic nephropathy (DN) is one of the most serious microvascular complications and the leading causes of death in diabetes mellitus (DM). To find biomarkers for prognosing the occurrence and development of DN has significant clinical value for its prevention, diagnosis, and treatment. In this study, a non-targeted cell metabolomics–based ultra-performance liquid chromatography coupled with quadrupole time of flight mass spectrometry and gas chromatography coupled with mass spectrometry was developed and performed the dynamic metabolic profiles of rat renal cells including renal tubular epithelial cells (NRK-52E) and glomerular mesangial cells (HBZY-1) in response to high glucose at time points of 12 h, 24 h, 36 h, and 48 h. Some potential biomarkers were then verified using clinical plasma samples collected from 55 healthy volunteers, 103 DM patients, and 57 DN patients. Statistical methods, such as principal component analysis and partial least squares to latent structure-discriminant analysis were recruited for data analyses. As a result, palmitic acid and linoleic acid (all-cis-9,12) were the potential indicators for the occurrence and development of DN, and valine, leucine, and isoleucine could be used as the prospective biomarkers for DM. In addition, rise and fall of leucine and isoleucine levels in plasma could be used for prognosing DN in DM patients. Through this study, we established a novel non-targeted cell dynamic metabolomics platform and identified potential biomarkers that may be applied for the diagnosis and prognosis of DM and DN.

## Introduction

Diabetic nephropathy (DN), with the characteristic of proteinuria, is one of the most serious microvascular complications in diabetes mellitus (DM) (Aldukhayel, 2017). The etiology and pathogenesis of DN are sophisticated and have not been fully elucidated yet. It is generally believed that, once formed, DN is difficult to be reversed. Thus, researchers have been committing to identify biologically specific biomarkers that can prognose DN in the stage of DM. Microalbuminuria is widely accepted as a clinically diagnostic indicator of early DN. However, relative studies have shown that only about 30% of DM patients with microalbuminuria will eventually develop into DN (Rossing et al., 2005). A considerable number of DM patients with microalbuminuria can actually change back to non-proteinuria state (Perkins et al., 2003). Moreover, when excess protein is present in urine, severe structural changes in the glomerular basement membrane will occur, leading to serious kidney damage (Fioretto et al., 1994). Therefore, exploration of laboratory biomarkers that are earlier than microalbuminuria or those appearing at the same time has remarkable significance for the early diagnosis and treatment of DN.

Metabolomics is an analytical technique for studying metabolic pathways by examining the overall changes of small metabolites in body fluids or tissues after exogenous stimulation or gene mutation in biological systems. Among them, biomarker discovery and determination of content changes over disease formation and progression are often referred to as dynamic metabolomics (You et al., 2018), the development of which provides us with an important research strategy for exploring biomarkers in the early formation of DN. In the *in vivo* metabolomics study of animals and humans, differences between species are often significant, and these are easily disturbed by factors such as diet, environment, age, and other diseases. Thus, enabling high-throughput identification of potential biomarkers is rather difficult. In contrast, *in vitro* cell metabolomics can be easily regulated in terms of experimental conditions, by which protein expression, cell signaling, and metabolism in discrete physiological processes could have higher repeatability than animal or human experiments. *In vitro* cellular experiments also require fewer sample sizes, which can reduce the experimental costs. Cells are normally more tolerant to unfavorable environments, hence generation of more abundant metabolome information. Metabolomics studies of cell samples can dynamically monitor changes of metabolites during disease progression (Zhang et al., 2013; Acunha et al., 2018). Therefore, the use of cellular dynamic metabolomics, combined with multiple statistical analysis methods, to investigate the dynamic changes of potential biomarkers in the process of cell metabolism under normal conditions and pathological conditions becomes an important approach to explore the pathogenesis of diseases. Commonly used techniques in metabolomics include gas chromatography coupled with mass spectrometry (GC-MS), liquid chromatography tandem with mass spectrometry (LC-MS/MS), nuclear magnetic resonance (NMR), and capillary electrophoresis coupled with mass spectrometry (CE-MS), *etc*. (Chan et al., 2011). With the rapid development of high-resolution mass spectrometry, GC-MS and LC-MS/MS have become the mainstream technology in dynamic metabolomics (Smilde et al., 2010).

In DN, inflammatory lesions caused by high glucose, hemodynamic abnormalities, oxidative stress, and extracellular matrix accumulation can initially result in tubulointerstitial fibrosis and glomerulosclerosis, etc., which could then develop into kidney failure (Zheng et al., 2016). Mesangial cells, the main cells in the mesangial area, play an important role in maintaining the structural integrity of the glomerular microvascular bed, providing mesangial matrix homeostasis, and regulating glomerular filtration and can be selected as the major target cells in the study of DN (Qiu et al., 2015; Lu et al., 2018). On the other hand, in the process of tubulointerstitial fibrosis, the main effector cells are fibroblasts, and 36% of the newly added fibroblasts are derived from renal tubular epithelial cells (Forino et al., 2006; Li et al., 2015). Thus, we used two cell lines, rat renal tubular epithelial cells (NRK-52E), and glomerular mesangial cells (HBZY-1), as the objects in our study of dynamic metabolomics that are suitable to explore biomarkers in the occurrence and development of DN.

In this study, NRK-52E and HBZY-1 treated with high glucose were used as a model. Non-targeted metabolomics investigation of cells cultured *in vitro* at different time points were performed using ultra-performance liquid chromatography coupled with quadrupole time of flight mass spectrometry (UPLC-Q/TOF-MS/MS) and GC-MS. Moreover, in order to verify our initial screening results, clinical samples from healthy volunteers and DM and DN patients were used to quantify the levels of some identified biomarkers. According to our study, several novel biomarkers for prognosing DN in DM patients were identified, which may have the potential for further clinical applications.

## Materials and Methods

### Chemicals, Reagents, and Materials

HPLC-grade methanol, acetonitrile, formic acid, and chloroform were purchased from MREDA (MA, USA). Low glucose (5.56 mmol/L) and high glucose (25 mmol/L) Gibco^®^ Dulbecco’s modified Eagle medium (DEME) were purchased from Nanjing KeyGen Biotech Co., Ltd. (Nanjing, China). Fetal bovine serum was purchased from Zhejiang Tianhang Biotechnology Co., Ltd. (Hangzhou, China).

### Cell Culture

NRK-52E and HBZY-1 cells were purchased form Cell Bank of Chinese Academy of Science (Shanghai, China). They were initially cultured in low-dose glucose DEME medium including 10% fetal bovine serum (v/v), 100 U/ml penicillin, and 100 μg/ml streptomycin at 37°C with 5% CO_2_. When the cell density reached approximately 80% or 90% confluence, the original culture solution was discarded. Cells were washed twice with 2 ml phosphate-buffered saline (PBS) buffer. 2.5% (v/v) trypsin (Vicmed, China) is gently tilted to make the cells immersed in the solution for complete digestion. Cells were then centrifuged at 1,000 rpm for 3 min after adding 2 ml DEME medium, and supernatants were discarded. Cells were then resuspended in 8 ml DEME medium and transferred to 50-ml centrifuge tube. Cell density was adjusted to around 0.5×10^5^/ml to 1×10^5^/ml with low glucose DEME medium and mixed by gently blowing. Then, 2 ml of cell suspension were respectively added to each well of two six-well plates and placed in cell culture incubator (Thermo Scientific Forma 3111 Water Jacketed CO_2_ Incubator). After culturing for 12 h, the two plates were given high glucose (HG group, n = 6) and low glucose (LG group, n = 6) DMEM medium containing 1% (v/v) fetal bovine serum, respectively, which were continuously cultured for 12, 24, 36, and 48 h until cellular extracts were measured. Cell morphologies were observed under light microscope.

### Sample Preparation for Metabolomic Study

For LC-MS, cells were harvested at each time point and rinsed with normal saline for three times. Liquid nitrogen was then added to quench the cells immediately. Quenched cells were rinsed by methanol-water solution (4:1 v/v) and then transferred to 1.5-ml centrifuge tubes with repeated freezing and thawing for six times in liquid nitrogen and 37°C water bath. After centrifugation at 12,000 rpm for 2 min, 800 μl supernatant and 200 μl methanol-water solution (4:1 v/v) were mixed, which was then vortexed for 30 s and centrifuged at 12,000 rpm, 4°C for 10 min. Two-microliter sample was injected into UPLC-Q/TOF-MS for further analysis. For GC-MS, cells were quenched as described above. 800 μl of supernatant from cell extract was freeze-dried and then mixed with 50 μl methoxyamine hydrochloride (MOX) pyridine solution (15 mg/ml). After 60°C oil bath for 2 h, 100 μl *N,O*-bis(trimethylsilyl)trifluoroacetamide (BSTFA) [containing 1% trimethylchlorosilane (TMCS)] was then added to the mixed solution. After 1-min vortex and 60-min oil bath (65°C), the mixed solution was centrifuged at 10,000 rpm for 10min and then injected for GC-MS analysis. At each time point, there will be a set of quality control samples, which were obtained by mixing cell extracts from three-well low-dose glucose and three-well high-dose glucose DMEM media, respectively.

### LC-MS Metabolic Profiling Analysis

UPLC-Q/TOF-MS/MS analysis was carried out using a 1290 system from Agilent (Agilent Technologies, Santa Clara, CA, USA) coupled with a quadrupole-time-of-flight mass spectrometer Agilent 6550 equipped with an orthogonal electrospray ionization (ESI) source (Agilent Jet Stream, AJS). The metabolomic profiling analysis was performed on a Fortis column (2.1 × 100 mm, 1.7 µm) using 0.1% (v/v) formic acid water solution (A) and 0.1% (v/v) formic acid acetonitrile solution (B) as mobile phase with a gradient program as follows: 5–45% B in 0–3 min, 45–65% B in 3–10 min, 65–95% B in 10–12 min, and 95% B in 12–14 min. Before each run, columns were re-equilibrated for 11 min using the initial solvent composition. The flow rate was set constant at 0.3 ml/min, and the column temperature was maintained at 40°C for all separations. Two microliters of samples solution were injected for each analysis, and metabolic profiling analyses were achieved in positive mode. MS operation conditions were as follows: capillary voltages, 4,000 V; nebulizer pressure, 28 psi; nozzle voltage, 300 V; drying gas flow rate, 12 L/min, drying gas temperature, 350°C; sheath gas temperature, 400°C; and sheath gas flow, 12 L/min. All analyses were performed in full-scan mode, and acquisition mass range was from m/z 50 to m/z 1,000.

### GC-MS Metabolic Profiling Analysis

GC-MS analysis was performed using an Agilent 7890A GC system coupled to an Agilent 5975C single-quadrupole mass spectrometer equipped with electron ionization (EI). The metabolomic profiling analysis was performed on an Agilent HP-5MS-UI column (60 m× 0.25 mm i.d., liquid film thickness of 0.25 μm). Helium was used as carrier gas with a flow rate of 1.0 ml/min. The injector temperature was 280°C, and the prepared sample (1 μl) was injected in the splitless mode. The initial column temperature was maintained at 70°C for 1 min, then increased at a rate of 10°C/min to 150°C and held at this temperature for 2 min, then increased at a rate of 20°C/min to 300°C and held at this temperature for 6.5 min. The EI source energy was set at 70  eV, and full-scan range was form m/z 40 to m/z 600. The source temperature was set at 230°C. Mass spectral signals were recorded after a 5.5-min solvent delay to avoid derivatization interferences.

### Clinical Validation of Some Potential Biomarkers

#### Sample Collection

Three cohorts of adults were recruited from the 2^nd^ Affiliated Hospital of Xuzhou Medical University, which included 55 healthy volunteers (21 males and 34 females), 103 DM patients (54 males and 49 females), and 57 DN patients (25 males and 32 females), diagnosed by clinical physician by the fasting plasma glucose and urine protein levels according to WHO criteria. Other relevant diseases are excluded through the collection of medical history and routine laboratory biochemical tests, such as malignant tumors, active bleeding, peptic ulcers, and hepatic failure, *etc*. Biochemical indicators of DM and DN patients were checked on the test day by searching patient’s hospital admission number. Biochemical indicators of healthy volunteers (NC group) were obtained by looking up corresponding physical examination reports. Relevant information such as gender, age, systolic blood pressure, diastolic blood pressure, blood glucose (BG), high-density lipoprotein cholesterol (HDLc), low-density lipoprotein cholesterol (LDLc), triglyceride (TG), total cholesterol (TC), creatinine (CREA), and urea is thoroughly collected. For detailed analysis of biochemical indicators, please refer to **Supplementary Table S1**. During research, the medication or treatment of patients from the clinical physician were not intervened. 2 ml of EDTA-anticoagulated fasting whole-blood samples collected from the upper limb vein of the subjects were centrifuged at 3,000 rpm for 10 min, and supernatants were transferred to 2 ml Eppendorf (EP) tube, which were then transported to -80°C freezer on ice pack for storage. The study was approved by the Medical Ethics Committee of Xuzhou Medical University and its 2^nd^ affiliated hospital (Approval number: XYFY2016-KL088). All patients provided written informed consent.

The pre-column derivatization combined with GC-MS was used for the quantification of selected fatty acids and amino acids in human plasma referred to our previous method (Du et al., 2019) with a slight modification. The detailed characters of GC-MS were listed in the Supplementary Materials.

### MS Data Processing and Statistical Analysis

For cell metabolomics, raw LC-MS and GC-MS data were processed using Agilent Mass Hunter, GC-MSD Analysis, and ProFinder 6.0. Alignment of drift (by retention time and mass) and data filtering were performed with Mass Profiler Professional (MPP, version 14.5, Agilent Technologies). Principal component analysis (PCA) and partial least squares to latent structure–discriminant analysis (PLS-DA) were recruited by MPP for pattern discriminant analyses. Metabolites and structures of potential biomarkers were identified by using METLIN and NIST 11.0 database. A p-value <0.05 was considered significant to select metabolites.

For quantitative analysis of potential biomarkers in clinical samples, the general condition, biochemical data, and linear discriminant analysis (LDA) were processed by SPSS l6.0. CREA was expressed by mean (min-max). Other measurement data were assessed by mean ± standard deviation (SD). Gender statistics was applied to χ*^2^* test, and the remaining indicators were analyzed by one-way ANOVA.

## Results

In the present study, a multiplatform metabolomics study has been carried out using UHPLC-Q/TOF-MS/MS and GC-MS to investigate dynamic metabolite changes in renal cells in response to high glucose. Some potential biomarkers obtained in metabolomics were simply validated using clinical sample.

### Effects of HG Interventions on Cell Morphologies

NRK-52E and HBZY-1 cells were cultured as previously described and exposed to by HG and LG media for 12, 24, 36, and 48 h, respectively. Effects of glucose interventions on NRK-52E and HBZY-1 cell morphologies were studies at each time point. It was observed under the light microscope that there are no significant changes in the morphologies of NRK-52E and HBZY-1 cells in LG group, inferring that low glucose medium might not affect cell growth and metabolism. In contrast, shape distortion and cell debris were observed in HG groups, which suggests that the HG medium has effects on growth and metabolism of the two types of cells with a probable time-dependent manner (**Supplementary Figure S1** and **Figure S2**). However, further metabolomics methods should be used for validating these observations.

### Effects of HG and LG Interventions on Metabolic Profiles of NRK-52E and HBZY-1 Cells Based on Optimized UPLC-Q/TOF-MS or GC-MS

The QC data were illustrated in **Supplementary Figure S3** and **Figure S4**. The chromatograms showed that the overlap of six QC samples was good, and the variation of retention time and peak response was little, and 3D-PCA score plot showed that QC samples were centralized in the same quarter, which meant that the developed LC-MS and GC-MS methods were suitable for the metabolomics study of renal cells.

Typical LC-MS and GC-MS chromatograms of NRK-52E and HBZY-1 cells in response to LG and HG were shown in **Supplementary Figures S5**–**S8**. It can be seen that the metabolites of NRK-52E and HBZY-1 cells from HG and LG media at each time point have multiple differences. The results from unsupervised PCA (**Supplementary Figures S9**–**S12**) showed that the obvious separation trend between HG samples and LG samples could be found at every time points, which hinted that there were obvious difference between the metabolomic profiles of HG group and LG group.

**Figure 1:**
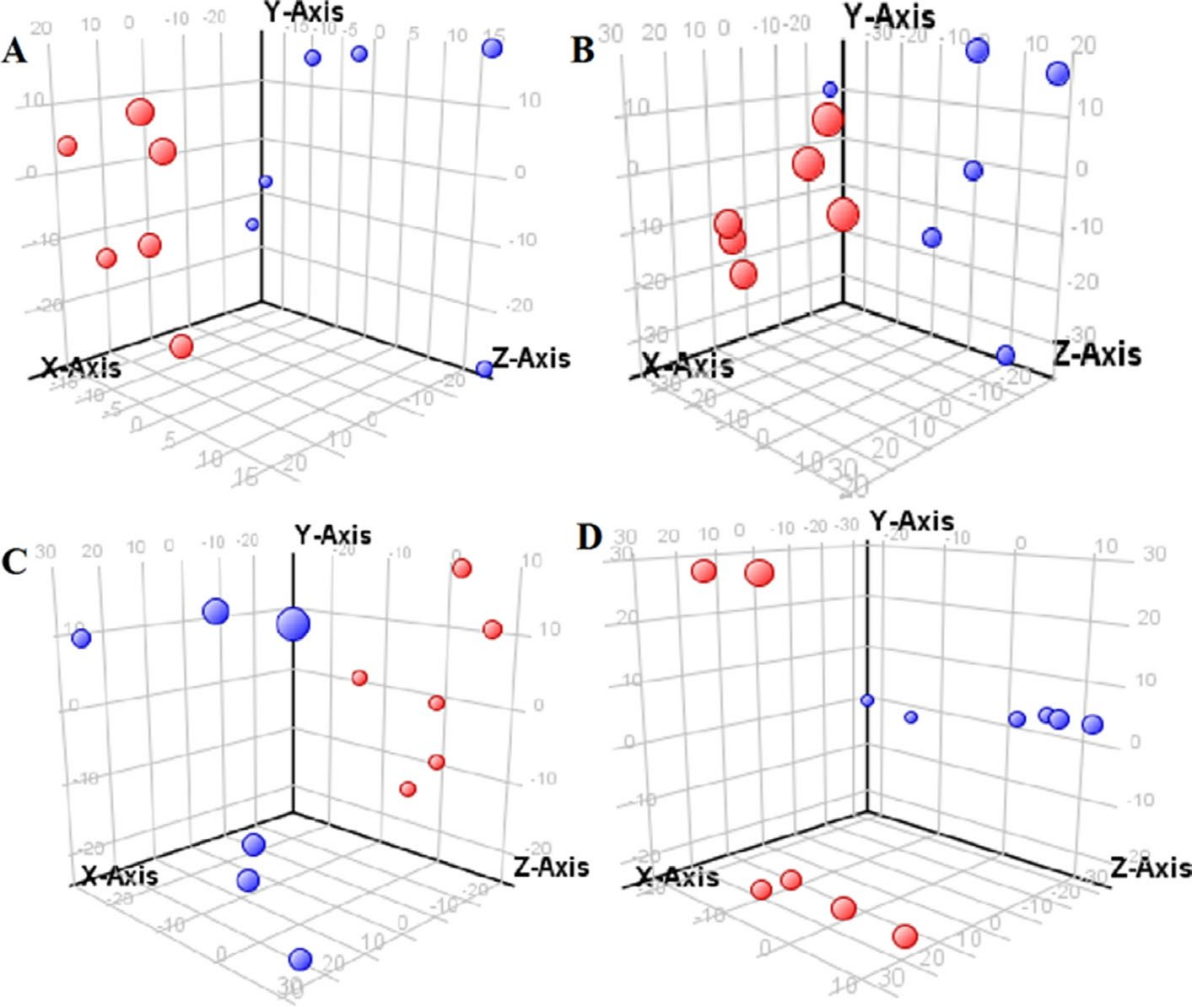
Partial least-square discriminant analysis score plot for NRK-52E cells in response to high glucose (HG, 25 mmol/L, red dot) and low glucose (LG, 5.56 mmol/L, green dot) for 12 h **(A)**, 24 h **(B)**, 36 h **(C)**, and 48 h **(D)** using LC-MS data.

**Figure 2:**
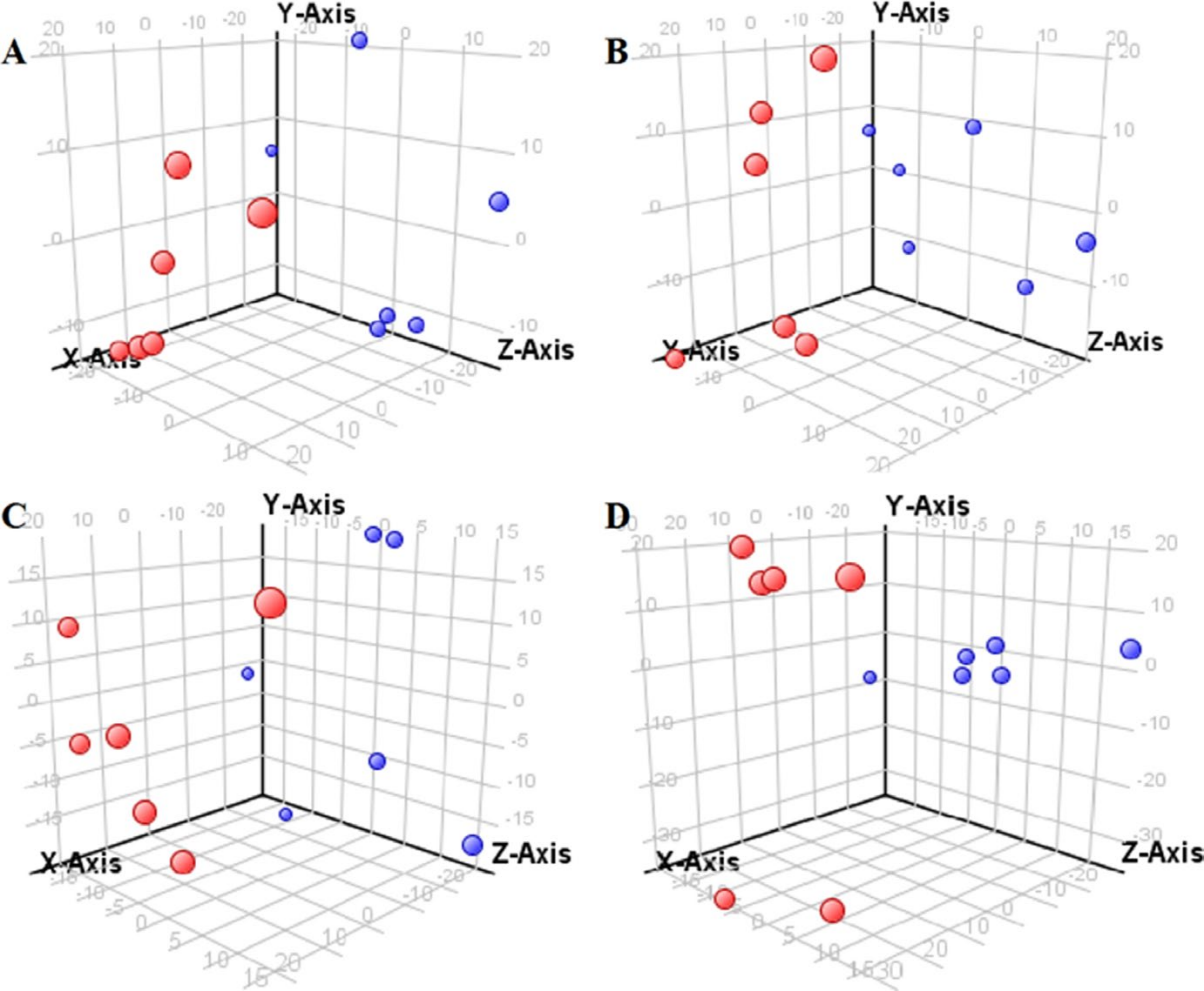
Partial least-square discriminant analysis score plot for HBZY-1 cells in response to high glucose (HG, 25 mmol/L, red dot) and low glucose (LG, 5.56 mmol/L, green dot) for 12 h **(A)**, 24 h **(B)**, 36 h **(C)**, and 48 h **(D)** using LC-MS data.

**Figure 3:**
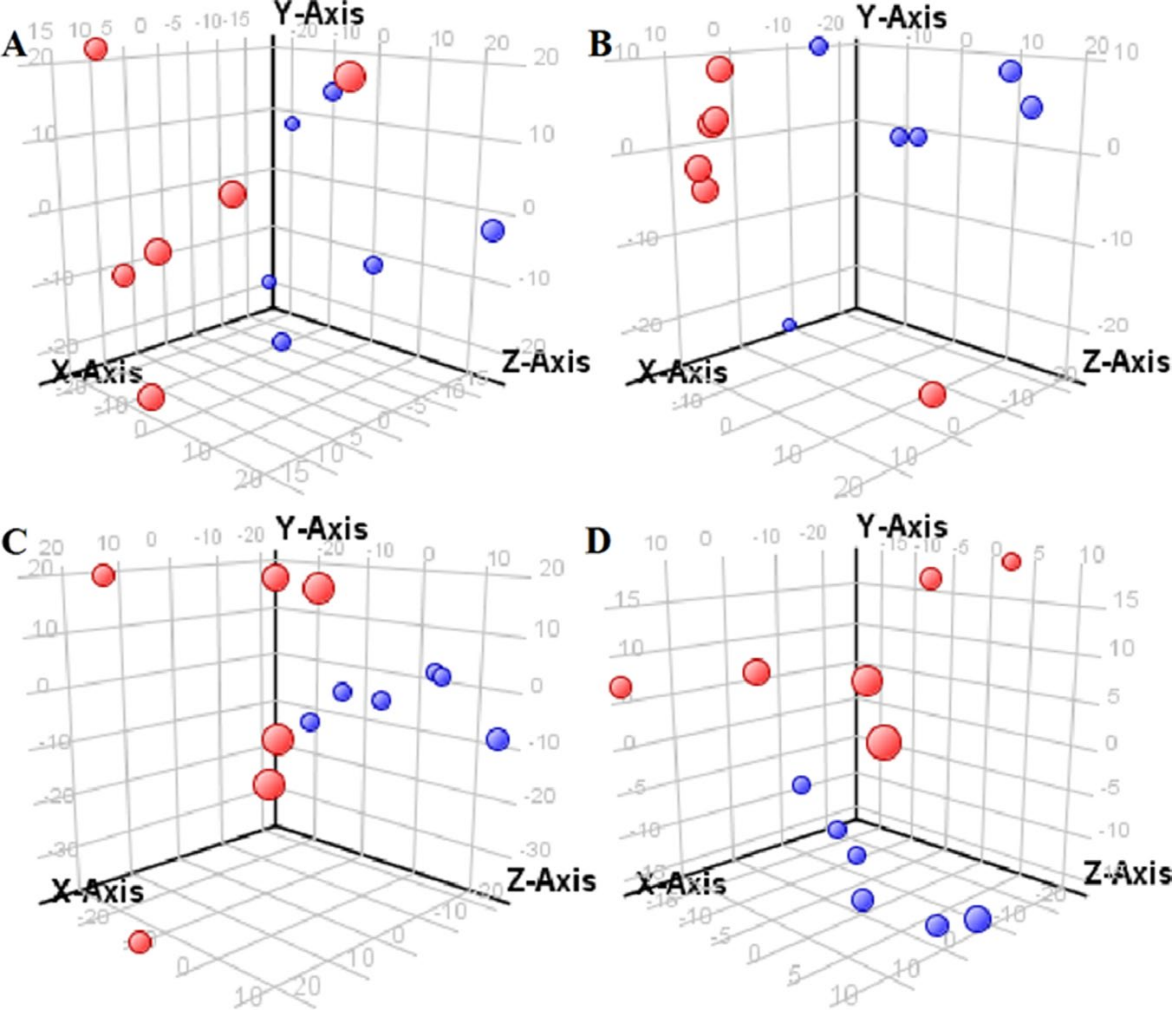
Partial least-square discriminant analysis score plot for NRK-52E cells in response to high glucose (HG, 25 mmol/L, red dot) and low glucose (LG, 5.56 mmol/L, green dot) for 12 h **(A)**, 24 h **(B)**, 36 h **(C)**, and 48 h **(D)** using GC-MS data.

**Figure 4:**
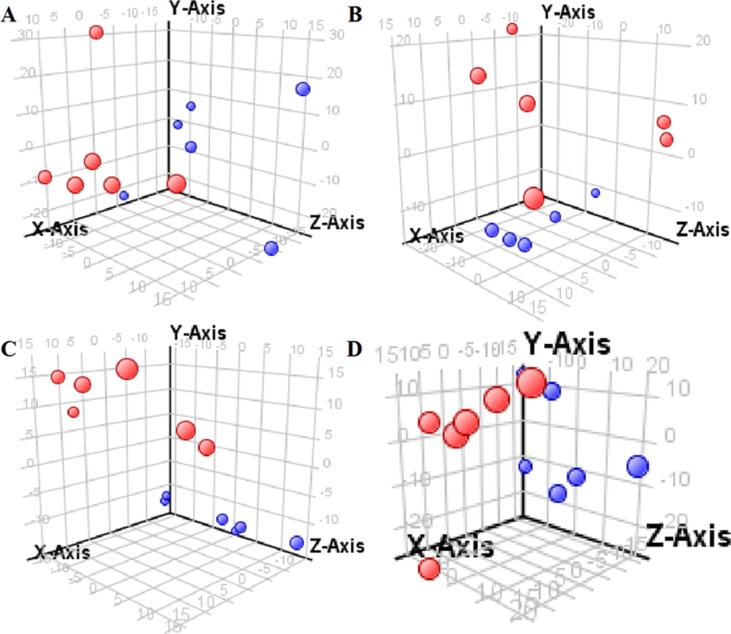
Partial least-square discriminant analysis score plot for HBZY-1 cells in response to high glucose (HG, 25 mmol/L, red dot) and low glucose (LG, 5.56 mmol/L, green dot) for 12 h **(A)**, 24 h **(B)**, 36 h **(C)**, and 48 h **(D)** using GC-MS data.

The supervised PLS-DA model can effectively eliminate the effects of individual outliers and be predicted to characterize the contribution of each sample. Thus, PLS-DA was used to analyze the data obtained by LC-MS or GC-MS in this study. From **Figures 1** to **4**, spatial distributions of LG and HG sample points were obviously separated at each time point, suggesting that there were significant differences in terms of metabolite profiles between the two groups. For the data obtained by LC-MS, the predictive ability scores of PLS-DA model whether for NRK-52E or HBZY-1 cells were all 100%; while for the data obtained by GC-MS, the predictive ability scores of PLS-DA model were greater than 83.3% for NRK-52E and 66.6% for HBZY-1 (**Supplementary Tables S2**–**S5**), which indicated that the PLS-DA model was stable and reliable. Statistically differential metabolites with persistent changes between LG group and HG group at 12, 24, 36, and 48 h in the two cell lines were listed in **Table 1**. It can be seen that the increased compounds in HG group compared with LG group in NRK-52E from 12 h were ceramide (Cer, d18:0/12:0), Cer (d18:0/14:0), and Cer (d18:0/16:0). Compound phosphatidylethanolamine (PE, 16:0/0:0) and glycine had a decreasing trend from 24 h while Cer (d20:0/16:0), and isoleucine tended to increase. The compounds that continuously declined were hypoxanthine, phosphatidylserine (PS, 14:0/12:0), PS (20:0/0:0), and PS (16:0/16:0) while leucine, palmitic acid, and phosphatidylglycerol (PG, 20:5/0:0) kept increasing. As for the HBZY-1 cells, compound LysoPE (0:0/22:6) decreased from 24 h while PG (20:5/0:0), leucine, and valine increased persistently. The compounds oleic acid, stearic acid, linoleic acid, PG (15:1/15:0), Cer (t18:0/16:0), Cer (d18:0/14:0), glucose, and galactose had a continuous rise from 36 h while sphingosine C16 and PE (19:0/0:0) kept declining. These results were partly consistent with the previous finding (Jensen et al., 2019).

**Table 1:** The statistically differential metabolites with persistent changes from NRK-52E and HBZY-1 cells in response to high glucose at different time points based on UHPLC-Q/TOF-MS or GC-MS.

Renal cell	Class, subclass	Metabolites	Significances (*p* value, *vs*. low glucose)				Analytical method
			12 h	24 h	36 h	48 h	
	**Amino acids**						
	Branched chain AA	Leucine	NS ^a^	NS	Up (*P* < 0.01)	Up (*P* < 0.05)	LC-MS
	Branched chain AA	Isoleucine	NS	Up (*P* < 0.01)	Up (*P* < 0.01)	Up (*P* < 0.01)	GC-MS
	AA	Glycine	NS	Down (*P* < 0.01)	Down (*P* < 0.01)	Down (*P* < 0.01)	GC-MS
	**Fatty acids**						
	FA (straight chain)	Palmitic acid	NS	NS	Up (*P* < 0.01)	Up (*P* < 0.05)	GC-MS
	**Imidazopyrimidines**						
	Purine derivatives	Hypoxanthine	NS	NS	Down (*P* < 0.01)	Down (*P* < 0.01)	LC-MS
NRK-52E	**Glycerophospholipids**						
	Glycerophosphoserine	PS (14:0/12:0)	NS	NS	Down (*P* < 0.01)	Down (*P* < 0.01)	LC-MS
	Glycerophosphoserine	PS (20:0/0:0)	NS	NS	Down (*P* < 0.01)	Down (*P* < 0.01)	LC-MS
	Glycerophosphoserine	PS (16:0/16:0)	NS	NS	Down (*P* < 0.01)	Down (*P* < 0.01)	LC-MS
	Diacylglycero phosphoglycerol	PG (20:5/0:0)	NS	NS	Up (*P* < 0.01)	Up (*P* < 0.01)	LC-MS
	Glycerophosphoethanolamine	PE (16:0/0:0)	NS	Down (*P* < 0.01)	Down (*P* < 0.01)	Down (*P* < 0.01)	LC-MS
	**Sphingolipids**						
	Ceramide	Cer (d20:0/16:0)	NS	Up (*P* < 0.01)	Up (*P* < 0.01)	Up (*P* < 0.01)	LC-MS
	Ceramide	Cer (d18:0/12:0)	Up (*P* <0.01)	Up (*P* < 0.01)	Up (*P* < 0.01)	Up (*P* < 0.01)	LC-MS
	Ceramide	Cer (d18:0/14:0)	Up (*P* <0.01)	Up (*P* < 0.01)	Up (*P* < 0.01)	Up (*P* < 0.01)	LC-MS
	Ceramide	Cer (d18:0/16:0)	Up (*P* <0.01)	Up (*P* < 0.01)	Up (*P* < 0.01)	Up (*P* < 0.01)	LC-MS
	**Amino acids (AA)**						
	Branched chain AA	Leucine	NS	Up (*P* < 0.01)	Up (*P* < 0.01)	Up (*P* < 0.01)	GC-MS
	Branched chain AA	Valine	NS	Up (*P* < 0.01)	Up (*P* < 0.01)	Up (*P* < 0.01)	GC-MS
HBZY-1	**Fatty acids (FA)**						
	FA (unsaturated)	Oleic acid	NS	NS	Up (*P* < 0.01)	Up (*P* < 0.01)	GC-MS
	FA (straight chain)	Stearic acid	NS	NS	Up (*P* < 0.01)	Up (*P* < 0.01)	GC-MS
	FA (unsaturated)	Linoleic acid	NS	NS	Up (*P* < 0.01)	Up (*P* < 0.01)	GC-MS
	**Glycerophospholipids**						
	Glycerophosphoethanolamine	PE (19:0/0:0)	NS	NS	Down (*P* < 0.01)	Down (*P* < 0.01)	LC-MS
	Diacylglycerophosphoglycerol	PG (15:1/15:0)	NS	NS	Up (*P* < 0.01)	Up (*P* < 0.01)	LC-MS
	Diacylglycerophosphoglycerol	PG (20:5/0:0)	NS	Up (*P* < 0.01)	Up (*P* < 0.01)	Up (*P* < 0.01)	LC-MS
	**Sphingolipids**						
	Ceramide	Cer (t18:0/16:0)	NS	NS	Up (*P* < 0.01)	Up (*P* < 0.01)	LC-MS
	Ceramide	Cer (d18:0/14:0)	NS	NS	Up (*P* < 0.01)	Up (*P* < 0.01)	LC-MS
	Sphingoid base	Sphingosine C16	NS	NS	Down (*P* < 0.01)	Down (*P* < 0.01)	LC-MS
	**Lysophospholipid**	LysoPE (0:0/22:6)	NS	Down (*P* < 0.01)	Down (*P* < 0.01)	Down (*P* < 0.01)	LC-MS
	**Other metabolites**						
		Glucose	NS	NS	Up (*P* < 0.05)	Up (*P* < 0.05)	GC-MS
		Galactose	NS	NS	Up (*P* < 0.01)	Up (*P* < 0.01)	GC-MS

a NS: This metabolite was not statistically significant between HG group and LG group; PE: phosphatidylethanolamine; PG: phosphatidylglycerol; PS: phosphatidylserine.

### Clinical Verification of Biomarkers Identified in Cell Metabolomics

Because the quantitative method of potential biomarkers in human plasma was based on our previous method (Du et al., 2019), partial validation was performed, and validation data such as regression equation, correlation coefficient, linear range, and mass fraction corresponding to each fatty acid and amino acid were shown in **Supplementary Tables S6** and **S7**.

**Figure 5:**
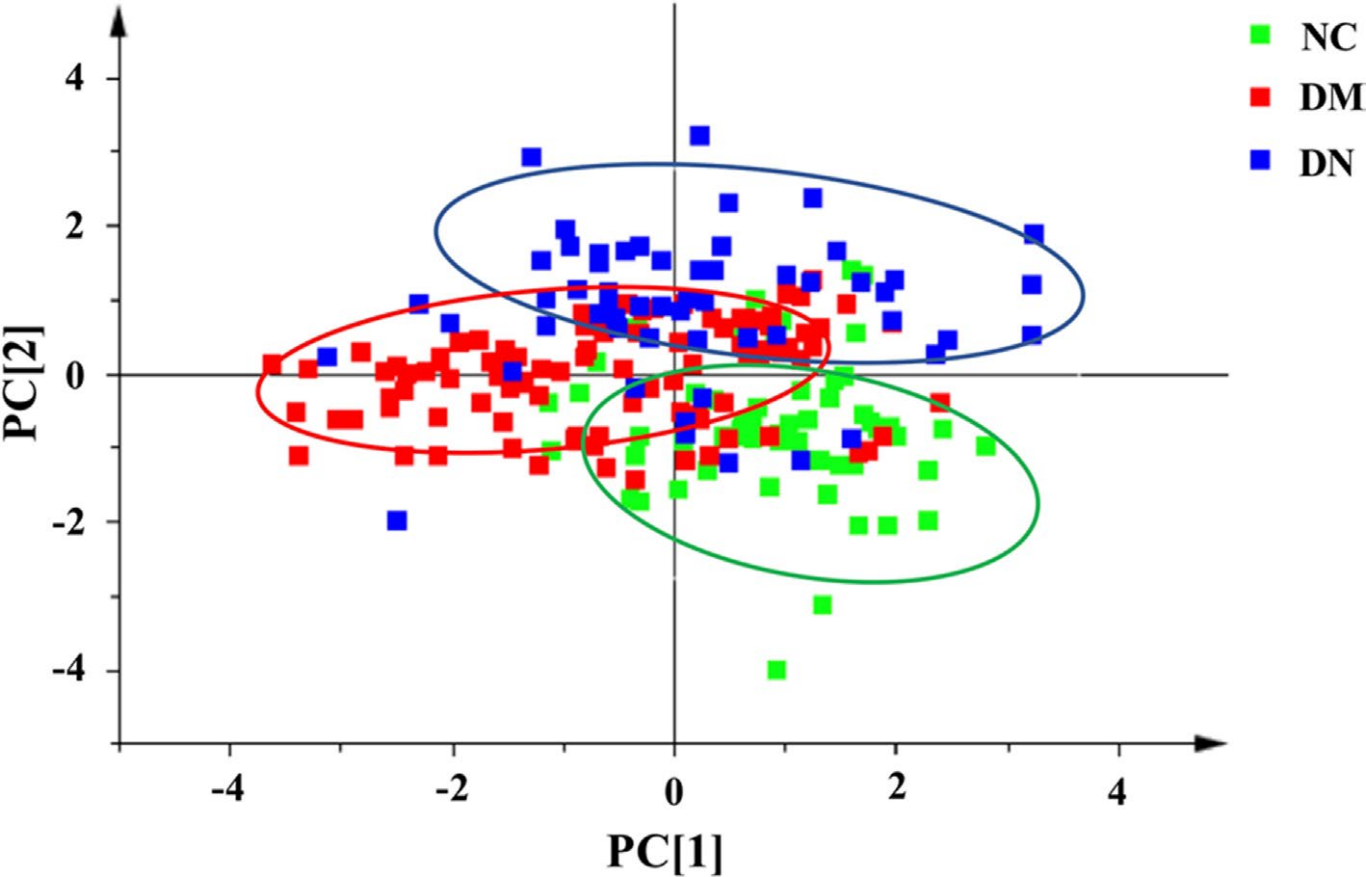
Linear discriminant analysis score plot of plasma samples from three group of subjects (NC: healthy control group; DM: diabetes mellitus group; DN: diabetic nephropathy group).

In order to effectively distinguish the three groups, data was analyzed using supervised LDA. Overall difference between the groups was distinguished by a linear combination of all compound characteristics, as shown in **Figure 5**. Each point in the figure represented a sample. The closer the sample points were, the closer the content of each compound was. The figure showed that the distribution of sample points in the NC, DM, and DN groups had a clear aggregation trend, while the sample points between the groups showed a good distinction.

The contents of selected potentially biomarkers in clinical plasma were shown in **Table 2**. From the results, we could find that compared with NC group, the DM group plasma content of stearic acid was significantly decreased while valine, leucine, and isoleucine were obviously increased; DN group plasma contents of stearic acid and linoleic acid were obviously reduced while palmitic acid and leucine were significantly enhanced higher than that of NC group, respectively. When compared with DM group, the plasma contents of palmitic acid in DN group was obviously increased while the plasma contents of stearic acid, valine, leucine, and isoleucine were significantly decreased. Moreover, persistent increasing or decreasing could be found for palmitic acid or stearic acid and linoleic acid from NC to DM and then DN, while first increment in DM and then decrement in DN happened for valine, leucine, and isoleucine.

**Table 2:** The concentrations of some metabolites in the plasma of subjects (mean ± SD, µg/ml).

Metabolites	NC (n = 55)	DM (n = 103)	DN (n = 57)
Palmitic acid (C16:0)	272.68 ± 111.81	309.55 ± 133.90	371.01 ± 107.63*^#^
Stearic acid (C18:0)	259.07 ± 69.63	207.01 ± 77.27*	166.98 ± 52.94*^#^
Oleic acid (cis-9-C18:1)	109.06 ± 36.66	111.10 ± 100.67	97.46 ± 57.41
Linoleic acid (all-cis-9,12-C18:2)	462.94 ± 129.75	402.56 ± 248.23	360.54 ± 171.86*
Glycine	17.92 ± 6.10	16.05 ± 5.02	16.33 ± 6.47
Valine	26.31 ± 3.88	29.89 ± 6.71*	24.82 ± 6.36^#^
Leucine	17.91 ± 2.88	21.96 ± 4.86*	20.33 ± 4.86*^#^
Isoleucine	7.46 ± 1.33	9.21 ± 2.54*	7.57 ± 1.86^#^

*P < 0.05 vs. NC; ^#^P < 0.05 vs. DM.

## Discussion

### Optimization of Sample Treatment and Analytical Method

It is essential to quench the cells during cell collection and metabolite extraction in order to prevent compositional changes of intracellular metabolites. The principle of cell quenching is to terminate all enzyme activities as quickly as possible because many metabolites can be degraded in a short time under the catalysis of enzymes. A common method for cell quenching is to rapidly change the temperature or pH value. Since the characteristics of the cell itself may affect the quenching efficiency and the rate of metabolite leakage (Wu and Li, 2013), it is necessary to select suitable quenching methods for specific types of cells, in our situation, NRK-52E and HBZY-1 cells. In this study, two methods, liquid nitrogen and -80°C extraction solvent, were investigated for quenching effects, respectively. After GC-MS and LC-Q/TOF-MS analyses, we considered that quenching by liquid nitrogen is better based on the number of peaks and variation of the response values.

Disruption of quenched cells is also important during metabolomics analysis. Usually, a variety of cell disruption methods are selectable depending on cell types or compounds. However, non-targeted metabolomics studies require that metabolites in the biological samples and the response values should meet certain requirements. For example, the extracted metabolite components should represent as much as possible the overall metabolite profile. Two cell disruption methods, sonication and repeated cycles of liquid nitrogen and 37°C water bath for three times, were recruited to investigate cell disruption efficiencies. Results showed that there was no significant difference between the two methods, but a large amount of heat was generated during cell disruption by sonication. Thus, repeatedly freezing and thawing in liquid nitrogen and 37°C water bath were used for this study. For the two analytical platforms, GC-MS and LC-Q/TOF-MS, this experiment examined the effects of methanol, methanol/water (4:1, v/v), methanol/water/chloroform (4:1:1, v/v/v), and acetonitrile for metabolite extraction efficiencies. It was concluded that, as the extraction solvent, methanol/water/chloroform (4:1:1, v/v/v) was better used for GC-MS analysis while LC-Q/TOF-MS analysis preferred to methanol/water (4:1, v/v). In addition, MOX and BSTFA were used as derivatization reagents. Temperature and dosage were optimized, respectively. The investigated dosages were 30 μl MOX and 50 μl BSTFA (containing 1% TMCS) and 50 μl MOX and 100 μl BSTFA (containing 1% TMCS) while reaction temperatures were 60°C and 65°C. After analysis, it was found that the derivatization conditions used in this experiment, which were 50 μl MOX, 100 μl BSTFA (containing 1% TMCS), and 65°C, were preferred.

This experiment also examined three chromatographic columns, which were Agilent DB-23 (60 m×0.25 mm×0.15 μm), Agilent HP-5MS (30 m×0.25 mm×0.25μm), and Agilent HP-5MS UI (60 m×0.25 mm×0.25 μm). Results of the analysis showed that HP-5MS was able to detect more metabolites and had better separation capacity among metabolites than the other two columns. Therefore, the HP-5MS column was used in this study. In order to make the small molecular metabolites in the cells have better resolution in GC-MS, we also investigated various heating procedures. Temperature program is described as below. Initial temperature was 70°C, which is maintained for 1 min, then increased at 5°C/min until 150°C, kept for 2 min, increased to 300°C at 20°C/min, and finally kept for 2 min. In order to make the small molecular metabolites in the cells have good resolution in LC-Q/TOF-MS, a variety of gradient elution procedures were investigated in this experiment, and the optimal procedure used in this study was finally obtained.

### Potential Biomarkers

DN is one of the major complications of DM, which is a group of metabolic syndromes characterized by gradual increase of BG level. The pathogenesis of DN has not yet been elucidated. It is generally believed that the pathogenesis of DN involves multiple factors such as hyperglycemia, hemodynamics disturbance, oxidative stress, and inflammatory responses (Xiu et al., 2017). In the process of progression from DM to DN, changing patterns of small molecular metabolites in the body will occur, which may play distinct roles in the occurrence and development of DN.

In this study, non-targeted cell metabolomics was first performed, and the obtained results suggested that the constant changes of small molecular metabolites such as lipids, fatty acids, amino acids, carbohydrates, and nucleosides were involved in the process. In those potential biomarkers, fatty acids and amino acids had attracted extensive attention and been proved to be closely related to DM or DN (Campion et al., 2017; Welsh et al., 2018). Thus, differential metabolites with persistent changes including fatty acids (palmitic acid, stearic acid, oleic acid, and linoleic acid) and amino acids (glycine, valine, leucine, and isoleucine) in cell metabolomics were further selected as target to validate their clinically significance in DM or DN patients. The results hinted that plasma contents of palmitic acid, stearic acid, linoleic acid, valine, leucine, and isoleucine were significantly changed in DM or DN patients. However, those biomarkers validated in the subject plasma may reflect not only renal damage but also together with other tissues, as non-renal cell types were not used as the control.

Studies have shown that non-esterified fatty acids have certain toxic effects on pancreatic β cells (Giacca et al., 2011), which can further lead to insulin resistance. Palmitic acid and stearic acid are saturated fatty acid that play harmful roles in human body (Rogiers, 1981). Carnitine, together with palmitic acid, can be transferred into mitochondria in the form of fatty acylcarnitine to complete β-oxidation. Insulin resistance may inhibit β-oxidation of fatty acids (Lu et al., 2016a), resulting in the accumulation of palmitic acid and stearic acid in DM or DN, which in latter causes kidney damage (Lim et al., 2010; Chu et al., 2013; Lu et al., 2016b). In our study, elevated palmitic acid and stearic acid in the renal cells induced by high glucose and increased palmitic acid in the plasma of DM and DN patients were observed, which was consistent with the previous findings (Chu et al., 2013; Lu et al., 2016a; Lu et al., 2016b; Du et al., 2019). However, the plasma level of stearic acid obtained in DM and DN patients was contrary to that in cell and previous study (Chu et al., 2013; Lu et al., 2016a; Lu et al., 2016b). We speculated that these results may be related to the diet and medicine of the subjects and may be accidental.

Linoleic acid is recognized as an essential fatty acid. Low linoleate intake is associated with DN (Dos Santos et al., 2018), and its metabolites including γ-linoleic acid, the hydroxyoctadecadienoic acid (HODEs), and oxo-octadecadienoic acids (oxo-ODEs) have potent biological effects (Bull et al., 2003; Itoh et al., 2008). For instance, the 9-HODE and 13-HODE and their alcohol dehydrogenase–dependent ketone metabolites, 9-oxo-ODE and 13-oxo-ODE, are potent PPAR-γ ligands (Bull et al., 2003; Itoh et al., 2008). Therefore, the DN-associated reduction in the all-cis-9,12 linoleic acid may reflect a reduction in cellular linoleate, and when the content of linoleic acid (all-cis-9,12) is lowered, the levels of these PPAR-γ ligands may also be reduced, aggravating insulin resistance and inflammatory reaction, eventually causing damage to the kidney. In addition, cytochrome-P450-dependent metabolism of linoleate has been shown to be modulated during the regulation of renal sodium handling (Dreisbach et al., 2008). Therefore, low level of linoleic acid in DM or DN may decrease renal Na^+^ excretion, which could itself promote renal damage. It is also possible that shunting of linoleate away from γ-linoleate production, and thus reducing availability of intracellular arachidonate, prostandoids, and other key lipid autocoids, may in and of itself promote renal damage, as supplementation with γ-linoleic acid has been shown to positively impact DN (Jamal, 1994) regardless of the mechanisms. These results suggest that linoleic acid (all-cis-9,12) levels may be an important biomarker during the development of DN.

Under normal conditions, the constant conversion between proteins and amino acids is in a state of equilibrium. The input and output rates of amino acids in blood are almost equal, thereby maintaining a stable amino acid concentration. During the development of DN, the metabolism of the body is disordered, and the amino acid level is bound to change. Valine, leucine, and isoleucine are all branched-chain amino acids (BCAA) that are essential in cellular metabolism. They are not only involved in protein synthesis, but also play an important role in glucose metabolism. Different pathological stages of DNs mirror changed metabolic statuses of BCAA. Studies have shown that, during insulin resistance and DM states, BCAA content increases (Gimble et al., 2010; Lynch and Adams, 2014), while in the early stage of chronic kidney disease, BCAA content decreases (Kumar et al., 2012). By monitoring the levels of leucine and isoleucine in plasma of T2DM patients by Raman spectroscopy, Birech et al. found that both compounds were significantly increased (Multhoff et al., 2017), which provide a basis for rapid screening and early intervention of T2DM patients. In addition, the causal relationship between BCAA and insulin resistance has attracted widespread attention during the development of DM. It was confirmed that, when the level of high-fat diet or insulin-like growth factor is low, protein decomposition increases, and BCAA level in the body rises. It was initially thought that enhanced BCAA leads to activation of the mTOR/S6K1 kinase signaling pathway and phosphorylation of serine residues on IRS-1, which further cause insulin resistance (Newgard et al., 2009). However, many studies now confirm that activation of the mTOR/S6K1 kinase signaling pathway is not a sufficient condition to cause insulin resistance. In contrast, it is believed that elevated levels of CBAA are caused by protein degradation due to insulin resistance. At present, the hypothesis about BCAA metabolic disorder mechanism has been widely recognized. In specificity, metabolic disorder of BCAA causes accumulation of toxic metabolites such as branched α-keto acid and acyl-CoA, resulting in mitochondrial damage in β-cells. In addition, stress kinase is also activated, ultimately causing insulin resistance and DM (Lynch and Adams, 2014). The results of this study showed that the levels of valine, leucine, and isoleucine in the plasma of DM patients were higher than those of the NC group, which is consistent with the above theory. In the DN state, when the kidney is damaged, the protein in the body is elevated by the efflux of the kidney, breaking the balance between its synthesis and decomposition. Levels of valine, leucine, and isoleucine are all reduced. Therefore, BCAA can be used as a biomarker for DM in the body, and up and down trends of BCAA level can also serve as an early warning for the occurrence of DN. Moreover, as the medication or treatment of the patients was not intervened during whole research, the results of potential biomarkers may hint that there were not obvious associations between the medication or treatment of patients and the metabolic pathway with related to those potential biomarkers.

## Conclusion

In this study, we established UPLC-Q/TOF-MS and GC-MS non-targeted cell dynamic metabolomics platforms for the initial exploration of potential biomarkers in the occurrence and development of DN. Through combined analysis of amino acids and fatty acids metabolites in clinical samples, we identified that (1) palmitic acid and linoleic acid (all-cis-9,12) can be used as potential biomarkers during the development of DN; (2) valine, leucine, and isoleucine can be considered as potential biomarkers of DM; and (3) fluctuation of valine, leucine, and isoleucine, that is, first increment and then decrement, may have an early warning effect on kidney damage in DM patients.

## Data Availability

All datasets generated for this study are included in the manuscript and/or the **Supplementary files**.

## Ethics Statement

This study was carried out in accordance with the recommendations of “Ethics committee of Xuzhou Medical University Affiliated Hospital” with written informed consent from all subjects. All subjects gave written informed consent in accordance with the Declaration of Helsinki. The protocol was approved by the “Ethics committee of Xuzhou Medical University Affiliated Hospital.”

## Author Contributions

LW, YD, B-JX, and D-QT participated in research design. LW, YD, B-JX, XD, Q-HL, Q-QZ, C-XW, SJ, and M-ZG performed the experiments and(or) data analysis. LW, YD, B-JX, Q-HL and D-QT contributed to the writing of the manuscript.

## Funding

This study was supported by Natural Science Foundation of Jiangsu Province (BK2011211 and BK20181147), the Program for Distinguished Talents of Six Domains in Jiangsu Province (2012-YY-013), Innovative and Entrepreneurial Talent Scheme of Jiangsu Province (2017), and Jiangsu University Natural Science Foundation of China (16KJA350001 and 16KJB180028).

## Conflicts of Interests Statement

The authors declare that the research was conducted in the absence of any commercial or financial relationships that could be construed as a potential conflict of interest.

## References

[B1] Acunha T. García-Cañas V. Valdés A. Cifuentes A. Simó C. (2018). Metabolomics study of early metabolic changes in hepatic HepaRG cells in response to rosemary diterpenes exposure. Anal. Chim. Acta 1037, 140–151. 10.1016/j.aca.2017.12.006 30292288

[B2] Aldukhayel A. (2017). Prevalence of diabetic nephropathy among type 2 diabetic patients in some of the Arab countries. Int. J. Health Sci. (Qassim) 11, 1–4. PMC5327670 28293155

[B3] Bull A. W. Steffensen K. R. Leers J. Rafter J. J. (2003). Activation of PPAR gamma in colon tumor cell lines by oxidized metabolites of linoleic acid, endogenous ligands for PPAR gamma. Carcinogenesis 24, 1717–1722. 10.1093/carcin/bgg131 12949056

[B4] Campion C. G. Sanchez-Ferras O. Batchu S. N. (2017). Potential role of serum and urinary biomarkers in diagnosis and prognosis of diabetic nephropathy. Can. J. Kidney Health Dis. 4, 2054358117705371. 10.1177/2054358117705371 28616250 PMC5461910

[B5] Chan E. C. Pasikanti K. K. Nicholson J. K. (2011). Global urinary metabolic profiling procedures using gas chromatography-mass spectrometry. Nat. Protoc. 6, 1483–1499. 10.1038/nprot.2011.375 21959233

[B6] Chu X. Liu L. Na L. Lu H. Li S. Li Y. (2013). Sterol regulatory element-binding protein-1c mediates increase of postprandial stearic acid, a potential target for improving insulin resistance, in hyperlipidemia. Diabetes 62, 561–571. 10.2337/db12-0139 22966071 PMC3554356

[B7] Dos Santos A. L. T. Duarte C. K. Santos M. Zoldan M. Almeida J. C. Gross J. L. (2018). Low linolenic and linoleic acid consumption are associated with chronic kidney disease in patients with type 2 diabetes. PLoS One 13, e0195249. 10.1371/journal.pone.0195249 30092058 PMC6084813

[B8] Dreisbach A. W. Rice J. C. Japa S. Newman J. W. Sigel A. Gill R. S. (2008). Salt loading increases urinary excretion of linoleic acid diols and triols in healthy human subjects. Hypertension 51, 755–761. 10.1161/HYPERTENSIONAHA.107.100123 18227407

[B9] Du Y. Xu B. J. Deng X. Wu X. W. Li Y. J. Wang S. R. (2019). Predictive metabolic signatures for the occurrence and development of diabetic nephropathy and the intervention of Ginkgo biloba leaves extract based on gas or liquid chromatography with mass spectrometry. J. Pharm. Biomed. Anal. 166, 30–39. 10.1016/j.jpba.2018.12.017 30599279

[B10] Fioretto P. Steffes M. W. Mauer M. (1994). Glomerular structure in nonproteinuric IDDM patients with various levels of albuminuria. Diabetes 43, 1358–1364. 10.2337/diab.43.11.1358 7926312

[B11] Forino M. Torregrossa R. Ceol M. Murer L. Della Vella M. Del Prete D. (2006). TGFbeta1 induces epithelial-mesenchymal transition, but not myofibroblast transdifferentiation of human kidney tubular epithelial cells in primary culture. Int. J. Exp. Pathol. 87, 197–208. 10.1111/j.1365-2613.2006.00479.x 16709228 PMC2517360

[B12] Giacca A. Xiao C. Oprescu A. I. Carpentier A. C. Lewis G. F. (2011). Lipid-induced pancreatic β-cell dysfunction: focus on *in vivo* studies. Am. J. Physiol. Endocrinol. Metab. 300, E255–E262. 10.1152/ajpendo.00416.2010 21119027

[B13] Gimble J. M. Fiehn O. Garvey W. T. Newman J. W. Lok K. H. Hoppel C. L. (2010). Plasma metabolomic profiles reflective of glucose homeostasis in non-diabetic and type 2 diabetic obese African-American women. PLoS One 5, e15234. 10.1371/journal.pone.0015234 21170321 PMC3000813

[B14] Itoh T. Fairall L. Amin K. Inaba Y. Szanto A. Balint B. L. (2008). Structural basis for the activation of PPARgamma by oxidized fatty acids. Nat. Struct. Mol. Biol. 15, 924–931. 10.1038/nsmb.1474 19172745 PMC2939985

[B15] Jamal G. A. (1994). The use of gamma linolenic acid in the prevention and treatment of diabetic neuropathy. Diabet. Med. 11, 145–149. 10.1111/j.1464-5491.1994.tb02010.x 8200197

[B16] Jensen P. N. Fretts A. M. Yu C. Hoofnagle A. N. Umans J. G. Howard B. V. (2019). Circulating sphingolipids, fasting glucose, and impaired fasting glucose: the Strong Heart Family Study. EBioMedicine 41, 44–49. 10.1016/j.ebiom.2018.12.046 30594552 PMC6444022

[B17] Kumar M. A. Bitla A. R. Raju K. V. Manohar S. M. Kumar V. S. Narasimha S. R. (2012). Branched chain amino acid profile in early chronic kidney disease. Saudi J. Kidney Dis. Transpl. 23, 1202–1207. 10.4103/1319-2442.103560 23168849

[B18] Li Y. Li Y. J. Li Z. Qiu J. Y. Zheng X. X. Bian T. T. (2015). Screening for potential bioactive components in Ginkgo biloba extract by the rat renal tubular epithelial cell extraction and LC-MS/MS. Comb. Chem. High Throughput Screen. 18, 514–523. 10.2174/1386207318666150430114022 25924659

[B19] Lim J. C. Lim S. K. Han H. J. Park S. H. (2010). Cannabinoid receptor 1 mediates palmitic acid-induced apoptosis *via* endoplasmic reticulum stress in human renal proximal tubular cells. J. Cell. Physiol. 225, 654–663. 10.1002/jcp.22255 20506110

[B20] Lu H. Hao L. Li S. Lin S. Lv L. Chen Y. (2016b). Elevated circulating stearic acid leads to a major lipotoxic effect on mouse pancreatic beta cells in hyperlipidaemia *via a* miR-34a-5p-mediated PERK/p53-dependent pathway. Diabetologia 59, 1247–1257. 10.1007/s00125-016-3900-0 26969487

[B21] Lu Q. Zhou Y. Hao M. Li C. Wang J. Shu F. (2018). The mTOR promotes oxidative stress-induced apoptosis of mesangial cells in diabetic nephropathy. Mol. Cell. Endocrinol. 473, 31–43. 10.1016/j.mce.2017.12.012 29277549

[B22] Lu Y. Wang Y. Ong C. N. Subramaniam T. Choi H. W. Yuan J. M. (2016a). Metabolic signatures and risk of type 2 diabetes in a Chinese population: an untargeted metabolomics study using both LC-MS and GC-MS. Diabetologia 59, 2349–2359. 10.1007/s00125-016-4069-2 27514531

[B23] Lynch C. J. Adams S. H. (2014). Branched-chain amino acids in metabolic signalling and insulin resistance. Nat. Rev. Endocrinol. 10, 723–736. 10.1038/nrendo.2014.171 25287287 PMC4424797

[B24] Multhoff G. Birech Z. Mwangi P. W. Bukachi F. Mandela K. M. (2017). Application of Raman spectroscopy in type 2 diabetes screening in blood using leucine and isoleucine amino-acids as biomarkers and in comparative anti-diabetic drugs efficacy studies. PLoS One 12, e0185130. 10.1371/journal.pone.0185130 28926628 PMC5605051

[B25] Newgard C. B. An J. Bain J. R. Muehlbauer M. J. Stevens R. D. Lien L. F. (2009). A branched-chain amino acid-related metabolic signature that differentiates obese and lean humans and contributes to insulin resistance. Cell Metab. 9, 311–326. 10.1016/j.cmet.2009.02.002 19356713 PMC3640280

[B26] Perkins B. A. Ficociello L. H. Silva K. H. Finkelstein D. M. Warram J. H. Krolewski A. S. (2003). Regression of microalbuminuria in type 1 diabetes. N. Engl. J. Med. 348, 2285–2293. 10.1056/NEJMoa021835 12788992

[B27] Qiu J. Y. Chen X. Zheng X. X. Jiang X. L. Yang D. Z. Yu Y. Y. (2015). Target cell extraction coupled with LC-MS/MS analysis for screening potential bioactive components in Ginkgo biloba extract with preventive effect against diabetic nephropathy. Biomed. Chromatogr. 29, 226–232. 10.1002/bmc.3264 24925151

[B28] Rogiers V. (1981). Long chain non-esterified fatty acid patterns in plasma of healthy children and young adults in relation to age and sex. J. Lipid. Res. 22, 1–6. 7217774

[B29] Rossing P. Hougaard P. Parving H. H. (2005). Progression of microalbuminuria in type 1 diabetes: ten-year prospective observational study. Kidney Int. 68, 1446–1450. 10.1111/j.1523-1755.2005.00556.x 16164620

[B30] Smilde A. K. Westerhuis J. A. Hoefsloot H. C. Bijlsma S. Rubingh C. M. Vis D. J. (2010). Dynamic metabolomic data analysis: a tutorial review. Metabolomics 6, 3–17. 10.1007/s11306-009-0191-1 20339444 PMC2834778

[B31] Welsh P. Rankin N. Li Q. Mark P. B. Würtz P. Ala-Korpela M. (2018). Circulating amino acids and the risk of macrovascular, microvascular and mortality outcomes in individuals with type 2 diabetes: results from the ADVANCE trial. Diabetologia 61, 1581–1591. 10.1007/s00125-018-4619-x 29728717 PMC6445481

[B32] Wu Y. Li L. (2013). Development of isotope labeling liquid chromatography-mass spectrometry for metabolic profiling of bacterial cells and its application for bacterial differentiation. Anal. Chem. 85, 5755–5763. 10.1021/ac400330z 23495969

[B33] Xiu Z. M. Wang L. P. Fu J. Xu J. Liu L. (2017). 1-Acetyl-5-phenyl-1H-pyrrol-3-ylacetate: an aldose reductase inhibitor for the treatment of diabetic nephropathy. Bioorg. Med. Chem. Lett. 27, 4482–4487. 10.1016/j.bmcl.2017.08.002 28802633

[B34] You R. Dai J. Zhang P. Barding G. A. Jr. Raftery D. (2018). Dynamic metabolic response to adriamycin-induced senescence in breast cancer cells. Metabolites 8, pii: E95. 10.3390/metabo8040095 30558288 PMC6315875

[B35] Zhang A. Sun H. Xu H. Qiu S. Wang X. (2013). Cell metabolomics. OMICS 17, 495–501. 10.1089/omi.2012.0090 23988149 PMC3783970

[B36] Zheng S. Powell D. W. Zheng F. Kantharidis P. Gnudi L. (2016). Diabetic nephropathy: proteinuria, inflammation, and fibrosis. J. Diabetes. Res. 2016, 5241549. 10.1155/2016/5241549 26881250 PMC4736592

