# Supplementary material for: Metabolomics Study of Metabolic Changes in Renal Cells in Response to High-Glucose Exposure Based on Liquid or Gas Chromatography Coupled With Mass Spectrometry
Source: Front Pharmacol. 2019 Aug 20;10:928. doi: 10.3389/fphar.2019.00928 (PMC6711339; doi:10.3389/fphar.2019.00928)
Supplement: Supplementary file 1 [file DataSheet_1.docx]

**SUPPLEMENTARY MATERIALS**

**Metabolomics study of dynamically metabolic changes in renal cells in response to high glucose exposure based on liquid or gas chromatography coupled with mass spectrometry**

Liang Wang ^1, 2, #^, Yan Du ^1, 3, #^, Bing-ju Xu ^1, #^, Xu Deng ^1^, Qing-hua Liu ^1^, Qiao-qiao Zhong ^1^, Chen-xiang Wang ^1^, Shuai Ji ^1, 3^, Meng-zhe Guo ^1, 3^, Dao-quan Tang ^1, 3, *^

^1^Jiangsu Key Laboratory of New Drug Research and Clinical Pharmacy, School of Pharmacy, Xuzhou Medical University, Xuzhou 221004, China

^2^Department of Bioinformatics, School of Medical Informatics, Xuzhou Medical University, Xuzhou 221004, China

^3^Deparment of Pharmaceutical Analysis, School of Pharmacy, Xuzhou Medical University, Xuzhou 221004, China

^3^These authors contribute equally to the study

* Corresponding author: [tangdq@xzhmu.edu.cn](mailto:tangdq@xzhmu.edu.cn) or [tdq993@hotmail.com](mailto:tdq993@hotmail.com)

**Quantitative measurements of selected fatty acids and amino acids in human plasma**

For fatty acids, 60 μL plasma was mixed with 0.5 mL NaOH-CH_3_OH solution (0.5 mol/L) with 10 sec vortex, which were then heated at 75°C in oil bath for 8 min and cooled to room temperature. Sample was mixed with 0.5 mL BF_3_-CH_3_OH (14% w/v) and heated at 80^o^C in oil bath for 5min. After cooling to room temperature, 1.0 mL of 34.7 μg/mL ethyl hexanoate (IS_1_) in n-hexane solution and 0.4 mL saturated sodium chloride solution were added to the sample and vortex for 3 min before centrifuging at 3000 rpm for 10 min. 1 μL of the supernatant was injected into GC-MS for further analysis. The GC-MS quantification was carried out on an Agilent DB-23 capillary column (60 m × 0.25 mm × 0.15 µm) with a temperature program as follows: The initial column temperature was maintained at 70 °C for 1 min, then increased at a rate of 15 °C/min to 178 °C and held at this temperature for 4 min, then increased at a rate of 4 °C/min to 186 °C, 1 °C/min to 190 °C and held at this temperature for 1 min, then increased at a rate of 15 °C/min to 220 °C and held at this temperature for 5 min. Helium was used as carrier gas with a flow rate of 1.0 mL/min. The injector temperature was 220 ℃ and the prepared sample was injected in the splitless mode. The EI source energy was set at 70 eV and all quantifications of analytes were performed in the selected ion monitoring (SIM) mode. The source and quadrupole temperature were respectively set at 230 ℃ and 150 ℃. The solvent delay time was 7.5 min.

For amino acids, 300 μL plasma was mixed with 300 μL deionized water, 100 μL of 116.7 μg/mL norvaline solution (IS_2_), 400 μL ethanol, 100 μL pyridine, and 50 μL ethyl chloroformate (ECF). The mixed sample was allowed to stand for 1 min before 10 sec vortex and 1 min sonication. 300 μL chloroform was then added with 30 s vortex and 5 min centrifugation at 3000 rpm. After that, 100 μL 7 mol/L NaOH solution was added to the supernatant to adjust pH. At the same time, 50 μL ECF was added, vortexed for 20 sec, sonicated for 1 min, vortexed for 30 s, and centrifuged at 3000 rpm for 10 min. The supernatant layer was removed, and the chloroform layer was transferred to a 1.5 mL EP tube. A small amount of anhydrous sodium sulfate was added to remove excess water. Samples were further centrifuged at 12000 rpm for 10 min. 1 μL of the supernatant was injected for GC-MS analysis. The GC-MS analysis was carried out on an Agilent DB-624 capillary column (60 m × 0.25 mm × 1.4 µm) with a temperature program as follows: the initial column temperature was maintained at 70 °C, then increased at a rate of 20 °C/min to 230 °C and held at this temperature for 15 min. Except the solvent delay time at 10 min, other chromatographic and MS parameters were the same as that of fatty acid quantification.

**Table S1** Baseline characteristics of subjects involved in the study.

| Baseline characteristics | NC (n=55) | DM (n=103) | DN (n=57) |
| --- | --- | --- | --- |
| Gender (Male) | 21 (38.2%) | 54 (52.4%) | 25 (43.9%) |
| Age (Year) | 50.4±11.8 | 60.4±11.9^*^ | 62.9±12.4^*^ |
| Body Weight (Kg) | 64.9±11.0 | 69.4±10.3 | 69.1±14.6 |
| Systolic BP (mmHg) | 126.8±19.4 | 133.1±18.4 | 137.9±22.2^*^ |
| Diastolic BP (mmHg) | 77.3±13.1 | 78.4±10.5 | 80.8±9.2 |
| HDLc (mmol/L) | 1.27±0.27 | 1.22±0.29 | 1.27±0.43 |
| LDLc (mmol/L) | 2.83±0.44 | 2.57±0.12 | 2.48±0.46 |
| TC (mmol/L) | 4.91±0.62 | 4.42±1.26 | 4.84±1.35 |
| TG (mmol/L) | 1.18±0.42 | 2.14±1.91^*^ | 2.36±1.89^*^ |
| BG (mmol/L) | 5.22±0.46 | 10.15±3.85^*^ | 9.62±4.83^*^ |
| CREA (μmol/L) | 61.64 (47-89) | 61.52 (29-440) | 113.94 (40-661)^*#^ |
| UREA (mmol/L) | 4.71±1.22 | 4.90±1.80 | 7.46±5.53^*#^ |

NC: normal control group; DM: diabetes mellitus group; DN: diabetic nephropathy group; HDLc: high-density lipoprotein cholesterol; LDLc: low-density lipoprotein cholesterol; TC: total cholesterol; TG: triglyceride; BG: blood glucose; CREA: creatinine; UREA: urea.

Data are presented by mean ± standard deviation (SD); CREA is calculated through mean (min-max). Gender statistics is applied to χ^2^ test and the remaining indicators are analysed by one-way ANOVA; ^*^P<0.05 vs NC; ^#^P<0.05 vs DM.

**Table S2** The predictive ability scores of PLS-DA model for the LC-MS data in NRK-52E cells.

|  | Group | Scores | | | |
| --- | --- | --- | --- | --- | --- |
|  |  | 12 h | 24 h | 36 h | 48 h |
| Training set | LG | 100.0% | 100.0% | 100.0% | 100.0% |
|  | HG | 100.0% | 100.0% | 100.0% | 100.0% |
| Validation set | LG | 100.0% | 100.0% | 100.0% | 100.0% |
|  | HG | 100.0% | 100.0% | 100.0% | 100.0% |

**Table S3** The predictive ability scores of PLS-DA model for the LC-MS data in HBZY-1 cells.

|  | Group | Scores | | | |
| --- | --- | --- | --- | --- | --- |
|  |  | 12 h | 24 h | 36 h | 48 h |
| Training set | LG | 100.0% | 100.0% | 100.0% | 100.0% |
|  | HG | 100.0% | 100.0% | 100.0% | 100.0% |
| Validation set | LG | 100.0% | 100.0% | 100.0% | 100.0% |
|  | HG | 100.0% | 100.0% | 100.0% | 100.0% |

**Table S4** The predictive ability scores of PLS-DA model for the GC-MS data in NRK-52E cells.

|  | Group | Scores | | | |
| --- | --- | --- | --- | --- | --- |
|  |  | 12 h | 24 h | 36 h | 48 h |
| Training set | LG | 100.0% | 100.0% | 100.0% | 100.0% |
|  | HG | 100.0% | 100.0% | 100.0% | 100.0% |
| Validation set | LG | 83.3% | 100.0% | 100.0% | 100.0% |
|  | HG | 83.3% | 83.3% | 100.0% | 100.0% |

**Table S5** The predictive ability scores of PLS-DA model for the GC-MS data in HBZY-1 cells.

|  | Group | Scores | | | |
| --- | --- | --- | --- | --- | --- |
|  |  | 12 h | 24 h | 36 h | 48 h |
| Training set | LG | 100.0% | 100.0% | 66.7% | 66.7% |
|  | HG | 100.0% | 100.0% | 100.0% | 83.3% |
| Validation set | LG | 83.3% | 100.0% | 100.0% | 100.0% |
|  | HG | 83.3% | 66.7% | 100.0% | 100.0% |

**Table S6** GC-MS ion information of fatty acids and amino acids in plasma.

| Compounds | Fragment Ions |
| --- | --- |
| Palmitic acid (C16:0) | 41.1, 43.1, 55.1, 69.0, 74.0^*^, 87.0, 143.1, 227.2, 270.1 |
| Stearic acid (C18:0) | 41.1, 43.1, 55.1, 74.0^*^, 87.0, 143.1, 255.2, 298.1 |
| Oleic acid (cis-9-C18:1) | 41.1, 43.1, 55.1^*^, 69.1, 83.0, 97.1, 222.2, 264.1, 296.1 |
| Linoleic acid (all-cis-9,12-C18:2) | 41.1, 43.1, 67.0^*^, 81.0, 95.1, 109.1, 263.1, 294.1 |
| IS_1_ | 41.0, 43.0, 57.1, 88.0^*^, 101.0, 157.0, 283.1, 326.4 |
| Glycine | 30.1, 45.1, 56.1, 74.1, 102.1^*^, 175.1, 207.2 |
| Valine | 30.1, 43.1, 55.1, 72.1, 102.1, 116.1, 144.2^*^, 207.1, 281.0 |
| Leucine | 30.1, 43.0, 58.1, 72.0, 86.2, 102.1, 158.2^*^, 207.1, 281.1 |
| Isoleucine | 30.1, 43.0, 58.1, 72.0, 86.2, 102.1, 158.2^*^, 207.1, 281.1 |
| IS_2_ | 30.1, 43.1, 55.1, 72.1, 102.1, 116.1, 144.2^*^, 207.1, 281.0 |

*Quantitative Ions

**Table S7** The regression equation, correlation coefficient (*r*) and linear ranges of fatty acids and amino acids in human plasma (6 calibration points, 5 curves).

| Compound | Regression Equation | *r* | Linear Range (μg/mL) |
| --- | --- | --- | --- |
| Palmitic acid (C16:0) | y=5.88×10^-4^x-0.2917 | 0.9982 | 0.0522-120 |
| Stearic acid (C18:0) | y=1.16×10^-4^x-0.0162 | 0.9998 | 0.0348-80 |
| Oleic acid (cis-9-C18:1) | y=5.80×10^-5^x-0.1154 | 0.9956 | 0.0174-40 |
| Linoleic acid (all-cis-9,12-C18:2) | y=2.80×10^-5^x-0.0529 | 0.9966 | 0.0174-40 |
| Glycine | y = 0.0209x + 0.0481 | 0.9990 | 1.73-165.63 |
| Valine | y = 0.0188x + 0.0372 | 0.9997 | 1.84-176.25 |
| Leucine | y = 0.0187x + 0.0346 | 0.9995 | 1.66-159.69 |
| Isoleucine | y = 0.0172x + 0.0143 | 1.0000 | 1.58-151.56 |

“y” means the ratios of the analyte peak area to the IS peak area, “x” represents the concentration of analyte.

**
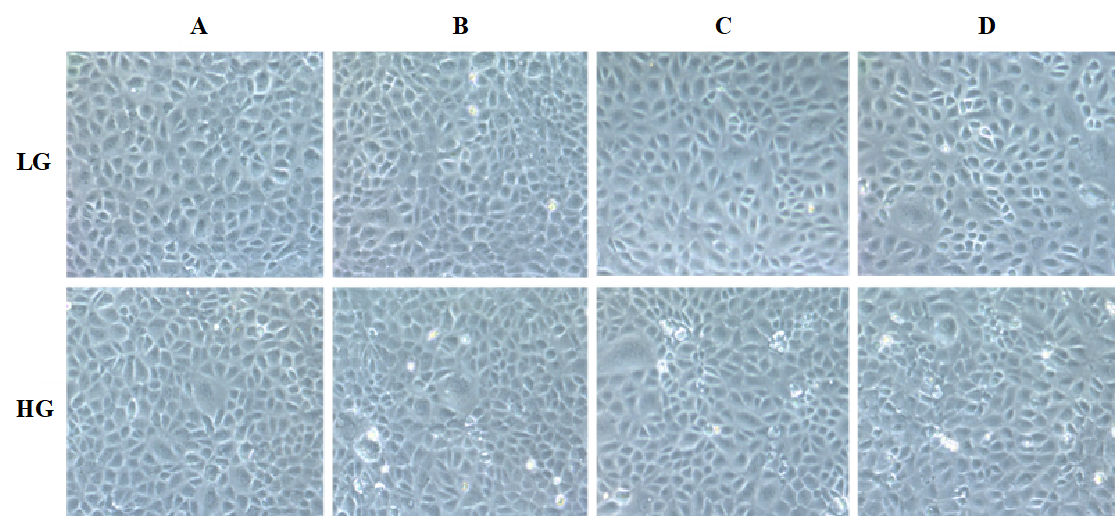
**

Figure S1 Effects of high glucose (HG, 25 mmol/L) and low glucose (LG, 5.56 mmol/L) interventions for 12 h (A), 24 h (B), 36 h (C) and 48 h (D) on NRK-52E cell morphology (light microscopy, ×100).


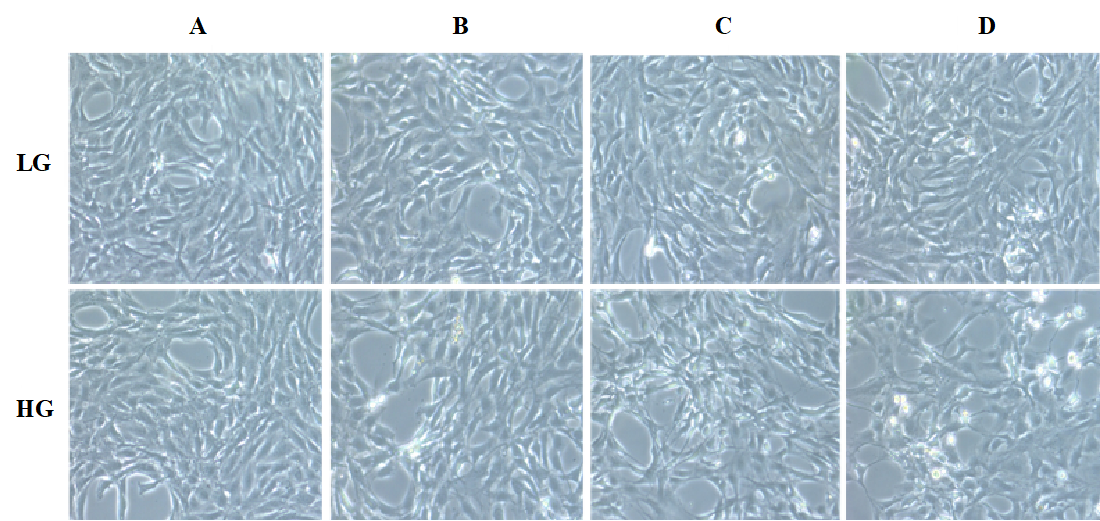


Figure S2 Effects of (HG, 25 mmol/L) and low glucose (LG, 5.56 mmol/L) interventions for 12 h (A), 24 h (B), 36 h (C) and 48 h (D) on HBZY-1 Cell Morphology (light microscopy, ×100).


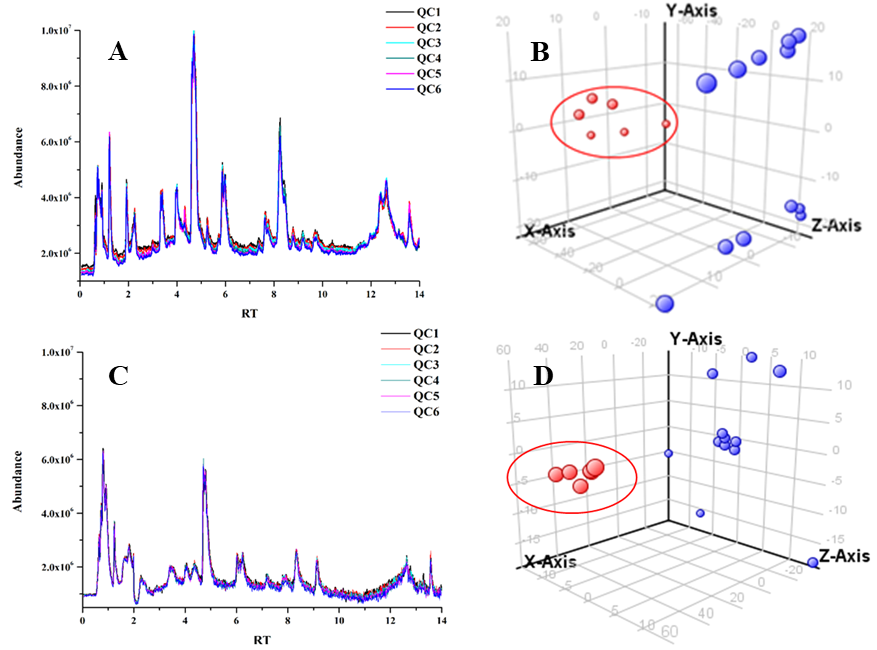


**Figure S3** The chromatograms of quality control samples for NRK-52E (A) and HBZY-1 cells (C) obtained from LC-MS and the 3D-PCA score plots of NRK-52E (B) and HBZY-1 cells (D). The red dots mean six quality control samples and the blue dots represent the cell samples from (LG, 5.56 mmol/ L) and high glucose (HG, 25 mmol/L) group.


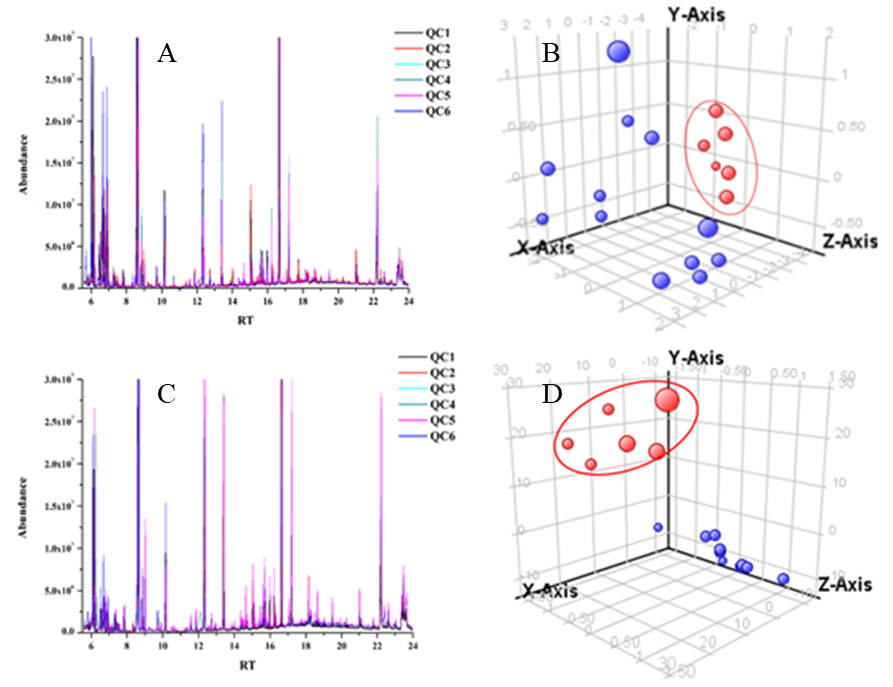


**Figure S4** The chromatograms of quality control samples for NRK-52E (A) and HBZY-1 cells (C) obtained from GC-MS and the 3D-PCA score plots of NRK-52E (B) and HBZY-1 cells (D). The red dots mean six quality control samples and the blue dots represent the cell samples from (LG, 5.56 mmol/L) and high glucose (HG, 25 mmol/L) group.


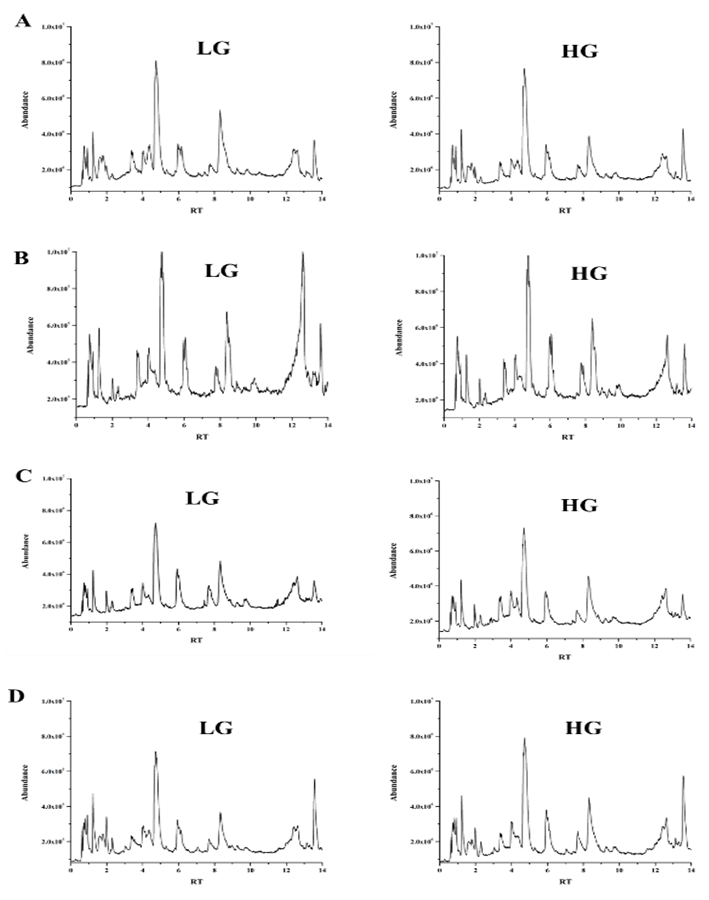


**Figure S5** Typical LC-MS chromatograms of cultured NRK-52E cells in response to low glucose (LG, 5.56 mmol/L) and high glucose (HG, 25 mmol/L) for 12 h (A), 24 h (B), 36 h (C) and 48 h (D).


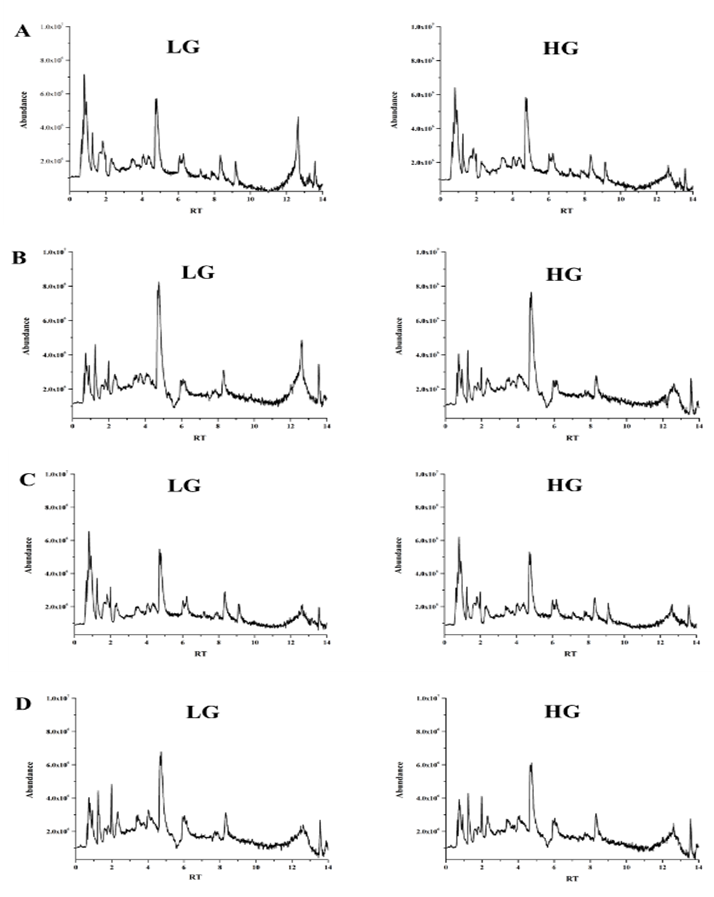


**Figure S6** Typical LC-MS chromatograms of cultured HBZY-1 cells in response to low glucose (LG, 5.56 mmol/L) and high glucose (HG, 25 mmol/L) for 12 h (A), 24 h (B), 36 h (C) and 48 h (D).


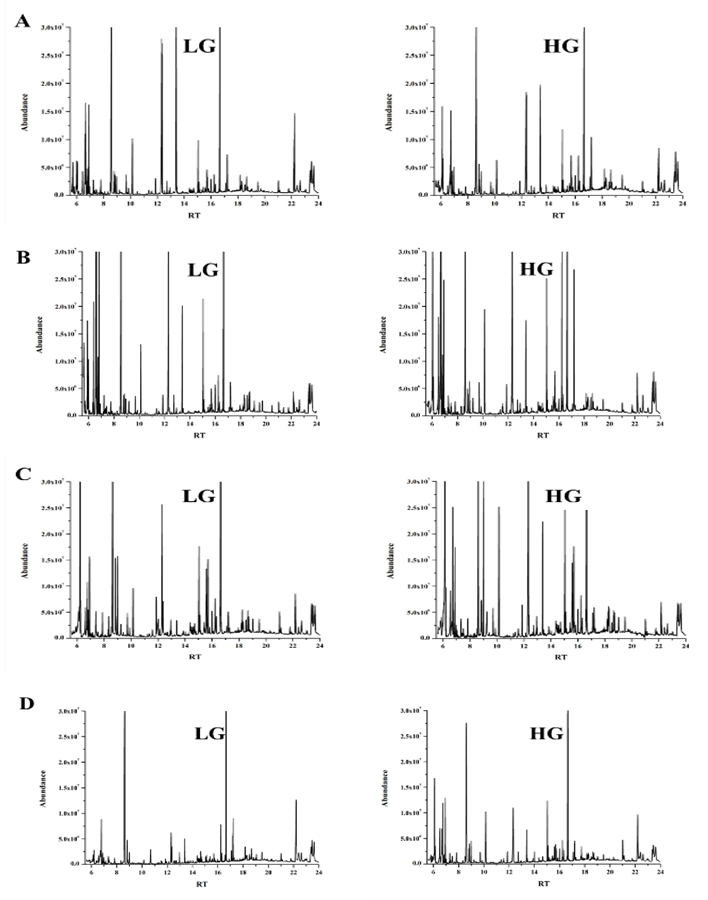


**Figure S7** Typical GC-MS chromatograms of cultured NRK-52E cells in response to low glucose (LG, 5.56 mmol/L) and high glucose (HG, 25 mmol/L) for 12 h (A), 24 h (B), 36 h (C) and 48 h (D).


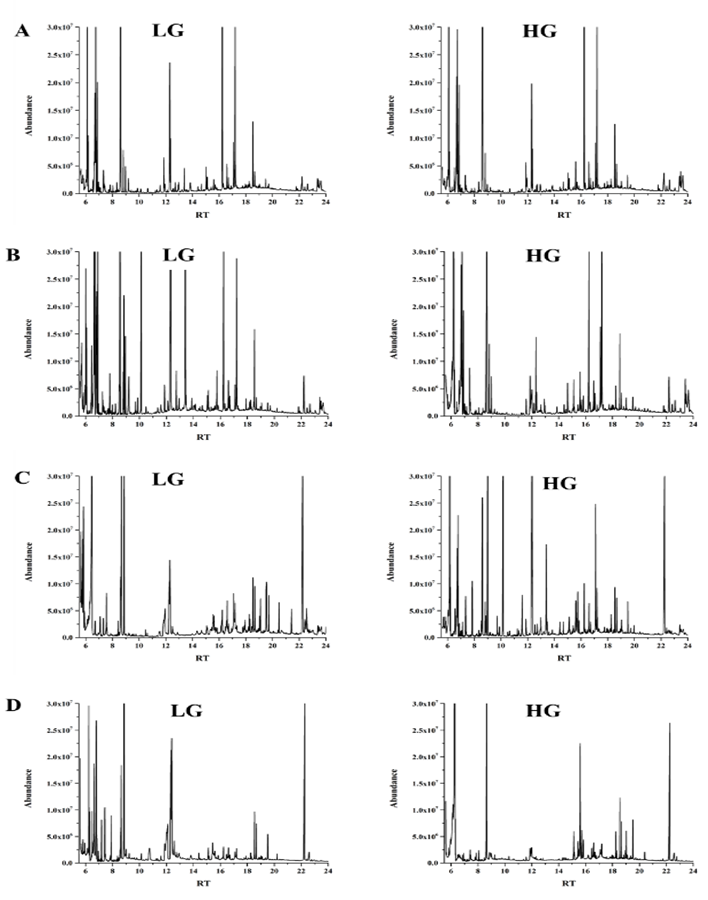


**Figure S8** Typical GC-MS chromatograms of cultured HBZY-1 cells in response to low glucose (LG, 5.56 mmol/L) and high glucose (HG, 25 mmol/L) for 12 h (A), 24 h (B), 36 h (C) and 48 h (D).


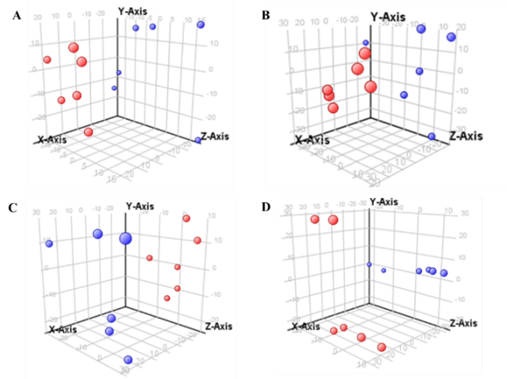


**Figure S9** The 3D-PCA score plots of NRK-52E cells in response to low glucose (LG, 5.56 mmol/L) and high glucose (HG, 25 mmol/L) for 12 h (A), 24 h (B), 36 h (C) and 48 h (D) analyzed by LC-MS. The red dots mean HG samples and the blue dots represent LG samples.


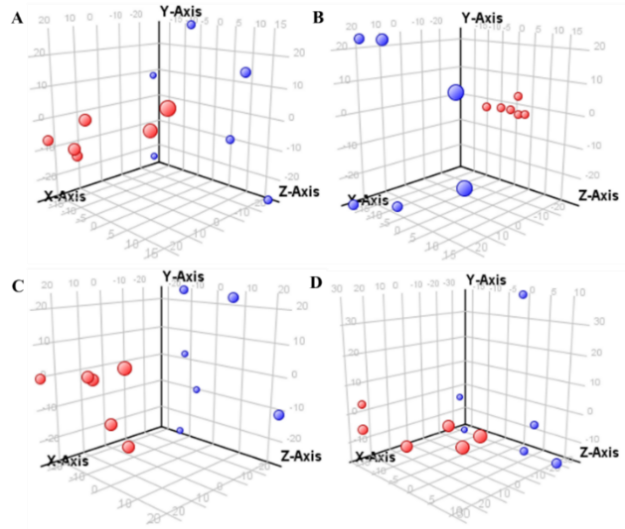


**Figure S10** The 3D-PCA score plots of HBZY-1 cells in response to low glucose (LG, 5.56 mmol/L) and high glucose (HG, 25 mmol/L) for 12 h (A), 24 h (B), 36 h (C) and 48 h (D) analyzed by LC-MS. The red dots mean HG samples and the blue dots represent LG samples.


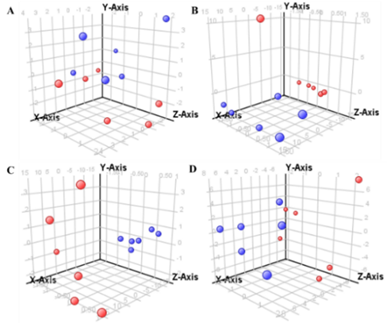


**Figure S11** The 3D-PCA score plots of NRK-52E cells in response to low glucose (LG, 5.56 mmol/L) and high glucose (HG, 25 mmol/L) for 12 h (A), 24 h (B), 36 h (C) and 48 h (D) analyzed by GC-MS. The red dots mean HG samples and the blue dots represent LG samples.


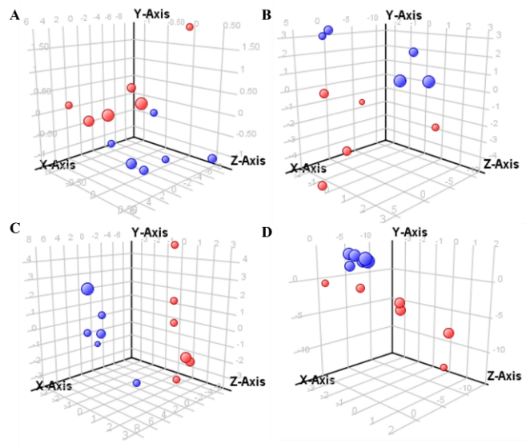


**Figure S12** The 3D-PCA score plots of HBZY-1 cells in response to low glucose (LG, 5.56 mmol/L) and high glucose (HG, 25 mmol/L) for 12 h (A), 24 h (B), 36 h (C) and 48 h (D) analyzed by GC-MS. The red dots mean HG samples and the blue dots represent LG samples.
